# Characterization and Isolation of the Major Biologically Active Metabolites Isolated from *Ficus retusa* and Their Synergistic Effect with Tetracycline against Certain Pathogenic-Resistant Bacteria

**DOI:** 10.3390/ph15121473

**Published:** 2022-11-26

**Authors:** Abdulwahab Alqahtani, Diaa A. Marrez, Mohamed Aleraky, Nada A. Fagir, Omaish Alqahtani, Samir Othman, Mohamed A. El Raey, Hany G. Attia

**Affiliations:** 1 Department of Pediatrics, College of Medicine, Najran University, Najran 1988, Saudi Arabia; 2 Food Toxicology and Contaminants Department, National Research Centre, Cairo 12622, Egypt; 3 Department of Clinical Pathology, College of Medicine, Najran University, Najran 1988, Saudi Arabia; 4 Department of Pharmacognosy, College of Pharmacy, Najran University, Najran 1988, Saudi Arabia; 5 Department of Pharmacognosy, Faculty of Pharmacy, 6th of October University, Giza 12585, Egypt; 6 Department of Phytochemistry and Plant Systematics, Pharmaceutical Industries Research Division, National Research Centre, Dokki, Cairo 12311, Egypt

**Keywords:** *Ficus* bark, catechin, chlorogenic acid, microbial resistance, tetracycline, synergistic activity

## Abstract

Globally, pathogenic microbes have reached a worrisome level of antibiotic resistance. Our work aims to identify and isolate the active components from the bioactive *Ficus retusa* bark extract and assess the potential synergistic activity of the most major compounds’ constituents with the antibiotic tetracycline against certain pathogenic bacterial strains. The phytochemical screening of an acetone extract of *F. retusa* bark using column chromatography led to the identification of 10 phenolic components. The synergistic interaction of catechin and chlorogenic acid as the most major compounds with tetracycline was evaluated by checkerboard assay followed by time-kill assay, against *Bacillus cereus, Staphylococcus aureus, Pseudomonas aeruginosa, Escherichia coli, Klebsiella pneumonia,* and *Salmonella typhi* with fraction inhibitory concentration index values (FICI) of 0.38, 0.43, 0.38, 0.38, 0.38, and 0.75 for catechin and 0.38, 0.65, 0.38, 0.63, 0.38, and 0.75 for chlorogenic acid. The combination of catechin and chlorogenic acid with tetracycline significantly enhanced antibacterial action against gram-positive and gram-negative microorganisms; therefore, catechin and chlorogenic acid combinations with tetracycline could be employed as innovative and safe antibiotics to combat microbial resistance.

## 1. Introduction 

It is a significant challenge for drug-discovery programs to stop the evolution of antibiotic resistance. The Centers for Disease Control and Prevention estimates that every year in the United States, at least two million people get serious infections caused by bacterial resistance to antibacterial drugs, and at least 23,000 people die as a result. Unfortunately, the World Bank predicts that by 2050, such bacterial-resistance diseases may harm the global economy as much as the 2008 financial crisis did [1,2]; therefore, it is critically desirable to develop new methods to manage bacterial infections. When employed alone or in combination with other antibacterial medicines, plant secondary metabolites (phytochemicals), such as phenolic compounds, have already shown their potential to be antibacterials [3]. There are, roughly, 800 species in the genus *Ficus*; collectively, they are referred to as fig trees. According to reports, *F. retusa* has significant levels of phenolic compounds and exhibits antioxidant, antibacterial, and anti-aging properties [4,5]. Tetracycline is one of the most often used antibiotics due to its wide range of activity, low toxicity, and affordable price [6,7]. Due to the rise in tetracycline-resistant isolates of clinically significant bacteria, the clinical use of tetracycline has been reduced in recent years [6]. The most well-studied and well-known mechanism of tetracycline resistance involves restricting tetracycline entrance to bacterial cell ribosomes. Tetracycline efflux is the process of pumping the antibiotic outside of the bacterial cell to lower intracellular tetracycline concentrations [8,9]. Despite the development of second, third, and fourth generations of tetracycline to combat bacterial resistance, assuming that enhancing the first-generation tetracycline’s efficacy and overcoming its resistance by mixing it with naturally occurring phenolic compounds may be more beneficial than using other generations with higher toxicity and mortality rates [6,10]. The F.D.A. informed medical professionals on 1 September 2010, that tigecycline (third-generation tetracycline) was significantly linked to an elevated risk for all-cause mortality in a pooled analysis of 13 clinical studies. For serious infections, the F.D.A. advises doctors to look into alternatives to tigecycline [11]. Acute hepatitis-like illness can occur within the first one to three months of treatment with the second-generation tetracycline, or a more pernicious chronic hepatitis with autoimmune symptoms can develop over the course of prolonged medication; additionally, utilizing the first generation of tetracycline on the Egyptian market is more affordable for patients than using the other generations, which are somewhat more expensive [12]. Our ongoing work aimed at phytochemical analysis of the bark of *F. nitidia*, then the phenolic metabolites from *F. retusa* acetone extract are isolated and identified in addition to evaluation of the synergistic efficacy of the most significant identified phenolic compounds with tetracycline against the common pathogenic antibiotic-resistant bacteria by means of checkerboard assay followed by time-kill assay.

## 2. Results 

### 2.1. Identification of Isolated Compounds from F. retusa Bark Acetone by N.M.R. Spectroscopic Analysis

Chromatographic and phytochemical investigation of *Ficus retusa* bark acetone led to the isolation and identification of 10 phenolic metabolites. All of these metabolites are known natural products that are identified using conventional spectroscopic and spectrometric analyses. Six compounds were isolated from *Ficus microcarpa* bark previously [13]. These were: (**1**) protocatechuic acid [14], (**2**) catechin [15,16], (**3**) chlorogenic acid, (**4**) methyl chlorogenate [14], (**5**) Procynandin B1 [17], and (**6**) procyanidin B3 [18]; moreover, four known compounds were isolated for the first time from *F. retusa* bark, namely: (**7**) catechin-3-*O*-glucoside [19], (**8)** isoferuyl quinic acid [20], (**9**) syringic acid [21], and (**10**) 7-*O*-Methyl-genistein [22].

**Figure 1:**
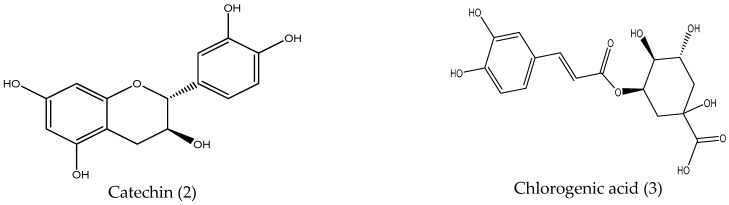
The chemical structure of catechin and chlorogenic acid.

Identification of catechin (2) Figure 1: ESI-MS: [M−H]–, 289 *m*/*z*, ^1^H-NMR (500 MHz, Acetone-d6 + D_2_O): δ ppm 4.49 (d, J = 7.6 Hz, H-2), 3.93 (m, H-3), 2.86 (dd, J = 5.35 and 16.05 Hz, H-4 eq), 2.47 (dd, J = 8.4 and 16.05 Hz, H-4 ax), 5.82 (d, J = 2 Hz, H-6), 6.75 (d, J = 2 Hz, H-8), 6.85 (d, J = 2 Hz, H-2′), 6.69 (d, J = 8 Hz, H-5′), 6.75 (dd, J = 8 and 2 Hz, H-6′).13C-NMR δ ppm 81.83 (C-2), 67.39 (C-3), 28.08 (C-4), 156.41 (C-5), 95.25 (C-6), 156.87 (C-7), 94.40 (C-8), 155.96 (C-9), 99.66 (C-10), 131.11 (C-1′), 114.46 (C-2′), 144.82 (C-3′), 144.91 (C-4′), 114.84 (C-5′), 119.14 (C-6′).

Identification of chlorogenic acid (3) Figure 1: ESI-MS: [M−H]^−^, 353 *m*/*z*, ^1^H-NMR (500 MHz, Acetone-d6 + D_2_O): δ ppm 1.88–2.18 (4H, m, H-2 and H-6), 5.28 (m, H-3), 3.65 (dd, J = 4.37, 13.5 Hz, H-4), 4.21 (m, H-5), 7.11 (d, J = 2 Hz, H-2′), 6.79 (d, J = 7.0, H-5′), 6.94 (dd, J = 7 and 2 Hz, H-6′), 7.50 (d, J = 16.05, H-7′) 6.24 (d, J = 16.05 Hz, H-8′).

### 2.2. Antibacterial Screening Test

#### 2.2.1. Antibacterial Activity of Tetracycline, Catechin, and Chlorogenic Acid

The antibacterial activities of tetracycline, catechin, and chlorogenic acid against six strains of pathogenic bacteria are illustrated in Table 1. Tetracycline showed zones of inhibition ranging from 17.3–26.8 mm, with the highest zone of inhibition at 26.8 mm against gram-negative *P*. *aeruginosa,* followed by 22.1 mm against *S*. *typhi*, while the lowest zone of inhibition was 17.3 mm against *E*. *coli*. Catechin and chlorogenic acid exhibited inhibition zones between 7.2–8.3 mm, with the highest zone of inhibition, 8.3 mm, against gram-positive *Staph*. *aureus* for catechin, and 8.2 mm against gram-negative *S*. *typhi* for chlorogenic acid, while the lowest zone of inhibition was 7.2 mm against gram-negative *P*. *aeruginosa.*

#### 2.2.2. Minimum Inhibitory Concentration and Synergy Interactions of Catechin with Tetracycline

The M.I.C. values of catechin and tetracycline in combination and individually were determined in Table 2. The M.I.C.s of catechin and tetracycline against tested pathogenic bacteria varied between 20 μg mL^−1^ and 3.0 mg mL^−1^. The lowest M.I.C. value exhibited by catechin was observed against *E*. *coli* (0.6 mg mL^−1^), while the highest M.I.C. value (3.0 mg ml^−1^) was observed against *P*. *aeruginosa*. The strong synergistic activity of the catechin and tetracycline combination is shown in Table 2. A significant reduction in M.I.C. values of catechin and tetracycline was recorded against the tested pathogenic bacteria. Fraction inhibitory concentration indices (FICI) showing synergy ranged from 0.38 and 0.43 with *B*. *cereus*, *E*. *coli*, *P*. *aeruginosa, S*. *typhi,* and *Staph*. *aureus*. An additive interaction was indicated with a FICI value of 0.75 with *K*. *pneumonia,* and no antagonism was recorded from the catechin and tetracycline combination.

#### 2.2.3. Minimum Inhibitory Concentration and the Synergy Interactions of Chlorogenic Acid

The M.I.C.s for chlorogenic acid and tetracycline alone and in combination are shown in Table 3. The M.I.C. values of tetracycline ranged from 20 to 54.2 μg mL^−1^ and the M.I.C. values of chlorogenic acid varied between 0.6–3.0 mg mL^−1^. The lowest M.I.C. value was observed by chlorogenic acid against *Staph. aureus* (0.6 mg mL^−1^) and the highest M.I.C. exhibited against *K*. *pneumonia* (3.0 mg mL^−1^).

The combination effect of chlorogenic acid and tetracycline was categorized into two classes, synergistic and additive effects, based on the FICI value (Table 3). The synergistic effect was observed against *B*. *cereus*, *E*. *coli*, and *S*. *typhi* with FICI 0.38, and an additive effect was seen against *P*. *aeruginosa, Staph*. *aureus*, and *K*. *pneumonia* with FICI 0.63, 0.65, and 0.75, respectively.

#### 2.2.4. Time-Kill Assay

**Figure 2:**
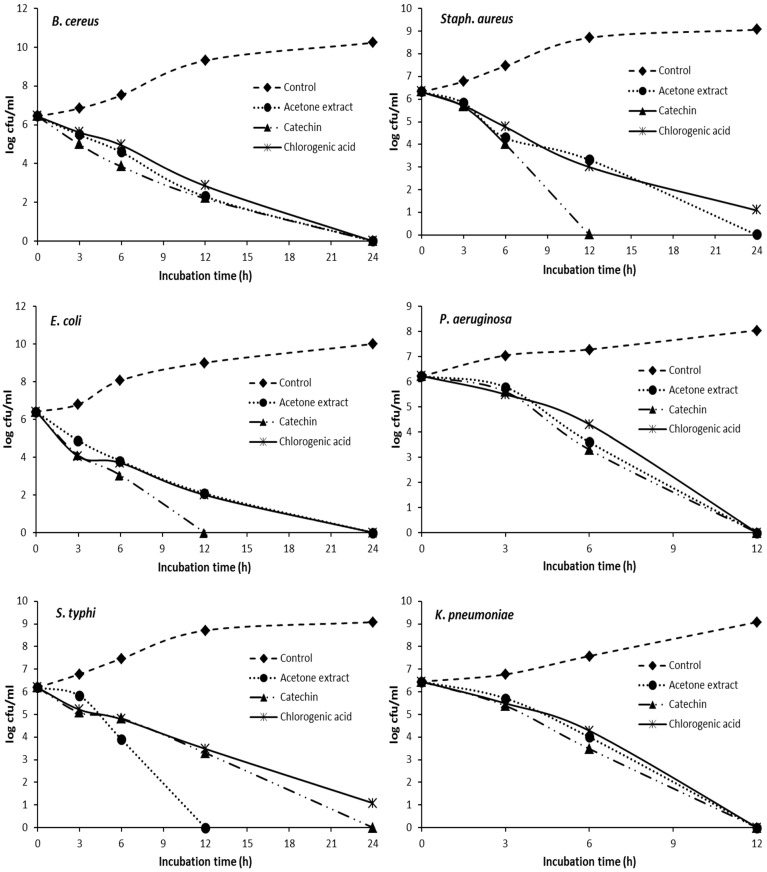
Time-kill data for a synergistic combination of tetracycline with acetone fraction, catechin, and chlorogenic acid against *B. cereus*, *Staph. aureus*, *E. coli*, *P. aeruginosa*, *S. typhi,* and *K. pneumoniae*.

To confirm the synergistic effect of catechin and chlorogenic acid with tetracycline against the tested pathogenic bacteria, a time-kill curve assay was conducted in Figure 2. The combination of 4.16, 4.16, and 13.55 μg mL^−1^ tetracycline and 150, 214.28, and 466 μg mL^−1^ catechin completely inhibited *E. coli*, *P*. *aeruginosa,* and *K*. *pneumoniae* growth within 12 h, respectively. While 2.5, 5.21, and 5.82 μg mL^−1^ tetracycline and 180, 182.5, and 91.25 μg mL^−1^ catechin required 24 h to completely inhibit the growth of *B*. *cereus*, *Staph. aureus* and *S*. *typhi*, respectively. The time-kill assay also confirmed the synergistic effect of chlorogenic acid and tetracycline combination Figure 2. The combination of 16.65 μg mL^−1^ tetracycline and 291.25 μg mL^−1^ chlorogenic acid as well as 13.55 μg mL^−1^ tetracycline and 1500 μg mL^−1^ demonstrated the synergistic effect by completely inhibiting the growth of *P*. *aeruginosa* and *K*. *pneumonia,* respectively, after 12 h incubation, while the synergistic effect of tetracycline and the chlorogenic acid combination was observed to completely inhibit the growth of *B*. *cereus* and *E. coli* within 24 h when combined with 2.5 and 4.16 μg mL^−1^ tetracycline with 417.5 μg mL^−1^ chlorogenic acid, respectively. In contrast, the combination of tetracycline and chlorogenic acid decreases the number of colony counts after 24 h in both *Staph. aureus* and *S*. *typhi* but not completely inhibiting bacterial growth.

## 3. Discussion

### 3.1. Antibacterial Activity of Tetracycline, Catechin, and Chlorogenic Acid

Chlorogenic acid and catechin are natural polyphenolic compounds. Natural phenolics have been linked to a variety of biological activities, including antimicrobial activity [23]. Polyphenolics affect the cell-wall proteins and the synthesis of nucleic acid [24]; however, other research implies that chlorogenic acid affects bacterial cell membranes, causing rupture of the membrane, which then allows macromolecules to leak into the cytoplasm and, ultimately, results in bacterial mortality [25,26]; moreover, in silico study revealed that phenolic compounds cause efflux pump inhibition [27].

As illustrated by the results in Table 1, the highest antibacterial activity of catechin was recorded against gram-positive *B*. *cereus* and *Staph*. *aureus* with 8.3 and 8.2 mm inhibition zones, respectively. At the same time, the highest zone of inhibition, 8.2 mm, was observed by chlorogenic acid against *S*. *typhi*. The antibacterial activities of catechin against gram-negative *E*. *coli*, *S*. *typhi*, *K*. *pneumonia*, and *P*. *aeruginosa* were less effective than those on gram-positive *Staph*. *aureus* and *B*. *cereus,* whereas the antibacterial activities of chlorogenic acid against gram-negative *E*. *coli* and *S*. *typhi* were more effective than those on gram-positive *Staph*. *aureus* and *B*. *cereus,* while the antibacterial activity of tetracycline was higher than that of catechin and chlorogenic acid against both gram-positive and gram-negative bacterial strains.

### 3.2. M.I.C. and Synergy Interactions of Catechin with Tetracycline

As illustrated by the results in Table 2, M.I.C. values achieved from catechin against the tested bacterial strains varied between 0.6 and 3.0 mg mL^−1^. The lowest M.I.C. value (0.6 mg mL^−1^) was observed against *E. coli,* while the highest M.I.C. value (3.0 mg mL^−1^) was recorded against *P. aeruginosa*. M.I.C. values of tetracycline ranged from 20 to 54.2 μg mL^−1^. The combinations between catechin and tetracycline against the mentioned bacterial strains were tested. M.I.C. values of tetracycline were significantly decreased when combined with catechin. This decrease depended on the tested bacterial strain; also, the M.I.C.s of catechin, when combined with tetracycline, were significantly decreased. The values of fraction inhibitory concentration index (FICI) acquired by the checkerboard assay were in the range of 0.38 to 0.43, indicating that all combinations studied had a synergistic effect (FICI < 0.5) in all strains except *K. pneumonia*, which had an additive effect (0.5 < ∑F.I.C. ≤ 1); hence, catechin boosted the antibacterial activity of tetracycline against *B. cereus, Staph. aureus, E. coli, P. aeruginosa,* and *S. typhi* with a synergistic effect (FICI values of 0.38, 0.43, 0.38, 0.38, and 0.38, respectively) and against *K. pneumonia* with an additive effect (FICI values of 0.75). Previously reported data confirmed the synergistic effects of catechin with antibiotics [28]. This synergistic activity could be explained due to the poly phenolic nature of catechin, which alters the membrane permeability (causing membrane rupture); hence, antibiotics such as tetracycline can penetrate the cell wall easily, leading to the death of both gram-positive and gram-negative bacteria [29,30].

### 3.3. M.I.C. and Synergy Interactions of Chlorogenic Acid with Tetracycline

As illustrated by the results in Table 3, M.I.C. values achieved from chlorogenic acid against the tested bacterial strains varied between 0.6 and 3.0 mg mL^−1^. The lowest M.I.C. value (0.6 mg mL^−1^) was observed against *Staph. aureus,* while the highest M.I.C. value (3.0 mg mL^−1^) was recorded against *K. pneumonia*. M.I.C. values of tetracycline ranged from 20 to 54.2 μg mL^−1^. The combinations between chlorogenic acid and tetracycline against the mentioned bacterial strains were tested. M.I.C. values of tetracycline were significantly decreased when combined with chlorogenic acid. This decrease depended on the tested bacterial strain; also, the M.I.C.s of chlorogenic acid, when combined with tetracycline, were significantly decreased. The value of the fraction inhibitory concentration index (FICI) acquired by the checkerboard assay was 0.38, indicating that all combinations studied had a synergistic effect (FICI < 0.5) in the strains *B. cereus, E. coli,* and *S. typhi,* while it had an additive effect (0.5 < ∑F.I.C. ≤ 1) in the strains *Staph. aureus, P. aeruginosa,* and *K. pneumonia;* hence, chlorogenic acid furthered the antibacterial activity of tetracycline against *B. cereus, E. coli,* and *S. typhi* with a synergistic effect (FICI values of 0.38, 0.38, and 0.38, respectively) and against *Staph. aureus, P. aeruginosa,* and *K. pneumonia* with an additive effect (FICI values of 0.65, 0.63, and 0.75, respectively).

### 3.4. Time-Kill Assay

In this study, *F*. *retusa* bark acetone extract, catechin, and chlorogenic acid inhibited the growth of all the tested bacteria that have been reported as pathogenic bacteria. The time-kill assay detected synergy against both gram-positive and gram-negative bacteria. Strong synergistic interaction with acetone extract and tetracycline against *S. typhi*, *P. aeruginosa,* and *K. pneumoniae* with complete inhibition was observed after 12 h. The synergy detected in acetone extract and tetracycline is not specific to any group of bacteria. Prinsloo and Meyer [31] reported that the synergistic effect of *Helichrysum sp*. crude extracts and antibiotics was attributed to the presence of a mixture of compounds that enhance the activity of different antibiotics. These findings explain why the synergistic effect of acetone extract with tetracycline was more than with catechin and chlorogenic acid.

## 4. Materials and Methods

### 4.1. Plant Material

The bark of *Ficus retusa* was collected from Obour City, Cairo, Egypt, in December 2018. Voucher specimen No. F101 was identified by Dr. Ibrahim El Garf, Prof. of Plant Taxonomy, Botany Department, Faculty of Science, Cairo University. The herbarium sample of the plant was deposited at the herbarium of the Pharmacognosy Dept., Faculty of Pharmacy, October 6 University.

### 4.2. Extraction and Isolation of the Compounds

The extraction method was conducted as mentioned by Embaby et al. 2021 [27]. Acetone extract 20 g was dissolved in water:methanol (90:10, *v*/*v*) and applied to a glass column packed with HP-20 dia-ion (500 g) and eluted with water followed by water/MeOH in order of decreasing polarity. The eluted fractions were visualized by Whatman 2D paper chromatography sheets No. 1 (2DPC), applied on Sephadex LH-20 column, and eluted by water:methanol (50:50, *v*/*v*) or butanol saturated with water (1:1 *v*/*v*, upper layer), then, the received sub-fractions were demonstrated by 2DPC, followed by application over Whatman paper chromatography 3MM for preparative isolation with solvent systems B.A.W. (butanol/acetic acid/water; 4:1:5) and/or 6% acetic acid to get 10 compounds (1–10). Final purification is conducted using Sephadex LH-20 column using methanol as eluent. The most major isolated compounds were catechin (144 mg) and chlorogenic acid (82 mg).

### 4.3. N.M.R. Spectroscopy

^1^H-nuclear magnetic resonance (N.M.R.) spectra were recorded on Jeol ECA-500 MHz N.M.R. spectrometer, and 125 MHz for ^13^C N.M.R. ^1^H chemical shifts (δ) were measured in ppm, relative to T.M.S. Mass spectrometry measurements were conducted using Thermo 3200QTRAP liquid chromatography (L.C.)/MS/MS System, and ultraviolet (U.V.) recordings were created on a Shimadzu UV-Visible-1601 spectrophotometer. Paper chromatographic (P.C.) analysis and preparative paper chromatography separation were carried out on Whatman filter paper sheets No. 1 and 3 MM papers, using solvent systems 15% HOAc and B.A.W. (n-BuOH-HOAc-H_2_O, 4:1:5, upper layer). HP-20 Dia-ion and Sephadex LH-20 were used.

### 4.4. Antibacterial Screening Test

#### 4.4.1. Disc Diffusion Assay

Each bacterial species’ 24 h-incubated nutritional agar slant had a loop full of the microbes injected into a 5 mL tube of tryptic soy broth (T.S.B.). The broth culture was then incubated for 2 to 6 h at 35 °C until it achieved 0.5 McFarland turbidity standards. The sensitivity test of catechin and chlorogenic acid was determined with different bacterial strains using the disc diffusion method by the Kirby–Bauer technique [32,33]. Dimethyl sulfoxide (DMSO) was used to dilute the samples to a concentration of 1 mg mL^−1^. Whatman No. 1 filter paper discs (6 mm) were filled with catechin and chlorogenic acid and fully dried. The discs were then put on the seeded plates. Tetracycline (500 μg mL^−1^) was represented as a positive control and DMSO was used as the negative control; after that, inoculated plates were incubated at 37 °C for 24 h. At the end of the incubation period, the zone of inhibition was determined and expressed as the clear zone diameter including the diameter of paper disc.

#### 4.4.2. Determination of Minimum Inhibitory Concentration (M.I.C.)

Minimal inhibitory concentration (M.I.C.) was determined for tetracycline, catechin, and chlorogenic acid by the micro broth dilution method [34,35]. Two-fold serial dilutions of catechin and chlorogenic acid were used, ranging from 10 mg mL^−1^ to 0.025 mg mL^−1^. The antibiotic used was tetracycline from 100 to 1 μg mL^−1^. Equal volumes of tested bacteria strains (10^5^ CFU mL^−1^) were inoculated into each well. M.I.C. values were determined as the lowest concentration that inhibited bacterial growth at 37 °C after 24 h incubation.

### 4.5. Synergistic Studies of Catechin and Chlorogenic Acid with Tetracycline

#### 4.5.1. Checkerboard Assay

Isobologram analysis was conducted by applying the checkerboard assay [36,37] to evaluate the presence of synergism or antagonism of catechin and chlorogenic acid with tetracycline. This method involves varying the concentrations of compounds and the antibiotic along different axes, ensuring that each well contains different combinations of the compounds and the antibiotic.

The analyses were performed in the microplates’ 96 wells. Bacteria were grown to reach optical density as of 2 × 10^8^ CFU mL^−1^. Five microliters of each bacterial strain was added into the well containing compounds, tetracycline, and M.H.B. The total volume in each well was 200 μL. The plates were incubated at 37 °C for 18 h. M.I.C. of the combination was measured as the lowest concentration, which completely inhibited bacterial growth. To evaluate the effect of the combinations, fractional inhibitory concentration (FICI) was calculated for each combination using the following formula:

FICA = MICA in combination/MICA alone;

FICB = MICB in combination/MICB alone;

FICI = FICA + FICB.

FICA is the F.I.C. of the tetracycline, MICA is the M.I.C. of the tetracycline, FICB is the F.I.C. of catechin or chlorogenic acid, and MICB is the M.I.C. of catechin or chlorogenic acid. The F.I.C. index is the F.I.C. added value of both the antibiotic and the samples. The interaction of the antibacterial combinations was evaluated by plotting an isobologram, as previously reported [38,39,40].

#### 4.5.2. Time-Kill Assay

Time-kill curve assays were achieved using the identified synergistic combinations of acetone extract, catechin, and chlorogenic acid with tetracycline against tested bacteria. The overnight growth plate was inoculated in sterile T.S.B. to achieve the approximate density of 0.5 McFarland standard. The suspension was diluted 1:10 in T.S.B. to obtain a standard inoculum of 1 × 10^6^ CFU/mL. One hundred μL of the diluted suspension was added to 0.9 mL of T.S.B. Double dilutions for each acetone extract, catechin, and chlorogenic acid in combination with tetracycline were prepared. Tubes containing the combinations were incubated at 35 °C for 24 h. From each tube, 100 μL of the sample was collected at 0, 4, 8, and 24 h, respectively, and inoculated to plate count media to determine the count of viable cells. Sterility control and growth control were included in each assay. The killing rate was determined by plotting viable colony counts (CFU/mL) against time. Synergy was defined as a ≥2 log10 CFU/mL decrease of viable count by the combination compared with the most active single agent.

### 4.6. Statistical Analysis

Results were subjected to a one-way analysis of variance (ANOVA) of the general linear model (G.L.M.) using the S.A.S. (1999) statistical package. The results were the average of three replicates (*p* ≤ 0.05). The dose-response values and time-kill data are presented as mean ± S.D. (Standard deviation). The analysis was performed in triplicate.

## 5. Conclusions

The combination of antibiotics with natural extracts is an effective way to reduce bacterial resistance to antibiotics and fight infections. Chlorogenic acid and catechin isolated from *F*. *retusa* bark are used in combination with tetracycline against different strains of drug-resistant bacteria. The current study showed a significant decrease in MIC values of tetracycline, chlorogenic acid, and catechin in combination, depending on the tested strains. Catechin showed synergistic effects when combined with tetracycline against *B*. *cereus*, *Staph*. *aureus*, *E*. *coli*, *P*. *aeruginosa,* and *S*. *typhi*, while the synergistic effects were observed against *B*. *cereus*, *E*. *coli,* and *S*. *typhi* in a case of chlorogenic and tetracycline combination, and the synergistic interaction was confirmed by time-killing assay. These results highlight the potential use of tetracycline with catechin or chlorogenic acid in combination therapies against multi-drug-resistant bacteria.

## Figures and Tables

**Table 1:** Antibacterial activity of tetracycline, catechin, and chlorogenic acid against the tested pathogenic bacterial strains.

Bacterial Strains	Inhibition Zone (mm) (Mean ± S.E.)			
	DMSO	Tetracycline	Catechin	Chlorogenic Acid
*Bacillus cereus*	0	21.4 ± 0.76	8.2 ± 0.28	7.5 ± 0.50
*Staphylococcus aureus*	0	20.1 ± 0.10	8.3 ± 0.28	7.8 ± 1.04
*Escherichia coli*	0	17.3 ± 1.42	7.2 ± 0.28	7.7 ± 1.15
*Salmonella typhi*	0	22.1 ± 1.15	7.8 ± 0.76	8.2 ± 0.28
*Pseudomonas aeruginosa*	0	26.8 ± 1.44	7.2 ± 0.29	7.2 ± 0.21
*Klebseilla pneumoniae*	0	20.8 ± 0.64	8.0 ± 0.28	7.3 ± 0.58

*n* = 3, *p* ˂ 0.05; S.E: standard error, different superscripts within the row (a, b, and c) are significantly different at the 5% level.

**Table 2:** M.I.C.s and F.I.C. indices of tetracycline (T.C.) in combination with catechin (C.T.) against the tested pathogenic bacterial strains.

Bacterial Strains	MIC_TC_ (µg mL^−1^)	MIC_CT_ (mg mL^−1^)	FIC_TC_	FIC_CT_	FIC Index	Interpretation
*Bacillus cereus*	20	0.72	0.13	0.25	0.38	S
*Staphylococcus aureus*	41.7	0.73	0.13	0.3	0.43	S
*Escherichia coli*	33.3	0.6	0.13	0.25	0.38	S
*Pseudomonas* *aeruginosa*	33.3	3.0	0.13	0.25	0.38	S
*Salmonella typhi*	23.3	0.73	0.25	0.13	0.38	S
*Klebseilla pneumoniae*	54.2	2.33	0.25	0.5	0.75	A

*n* = 3, *p* ˂ 0.05; S: synergistic effect; A: additive. The combination defined synergy if ∑F.I.C. ≤ 0.5, additive if 0.5 < ∑F.I.C. ≤ 1, indifference if 1 < ∑F.I.C. ≤ 4, and antagonism as ∑F.I.C. > 4.

**Table 3:** M.I.C.s and F.I.C. indices of tetracycline (T.C.) in combination with chlorogenic acid (C.A.) against six pathogenic bacterial strains.

Bacterial Strains	MIC_TC_ (µg mL^−1^)	MIC_CA_ (mg mL^−1^)	FIC_TC_	FIC_CT_	FIC Index	Interpretation
*Bacillus cereus*	20	1.67	0.13	0.25	0.38	S
*Staphylococcus aureus*	41.7	0.60	0.25	0.40	0.65	A
*Escherichia coli*	33.3	1.67	0.13	0.25	0.38	S
*Pseudomonas aeruginosa*	33.3	2.33	0.5	0.13	0.63	A
*Salmonella typhi*	23.3	0.87	0.25	0.13	0.38	S
*Klebseilla pneumoniae*	54.2	3.0	0.25	0.5	0.75	A

*n* = 3, *p* ˂ 0.05; S: synergistic effect; A: additive. The combination defined synergy if ∑F.I.C. ≤ 0.5, additive if 0.5 < ∑F.I.C. ≤ 1, indifference if 1 < ∑F.I.C. ≤ 4 and antagonism as ∑F.I.C. > 4.

## Data Availability

The data is contained within the article.
